# Mammalian animal models for dengue virus infection: a recent overview

**DOI:** 10.1007/s00705-021-05298-2

**Published:** 2021-11-10

**Authors:** Mohammad Enamul Hoque Kayesh, Kyoko Tsukiyama-Kohara

**Affiliations:** 1 Transboundary Animal Diseases Centre, Joint Faculty of Veterinary Medicine, Kagoshima University, Kagoshima, 890-0065 Japan; 2 Department of Microbiology and Public Health, Faculty of Animal Science and Veterinary Medicine, Patuakhali Science and Technology University, Barishal, 8210 Bangladesh

## Abstract

Dengue, a rapidly spreading mosquito-borne human viral disease caused by dengue virus (DENV), is a public health concern in tropical and subtropical areas due to its expanding geographical range. DENV can cause a wide spectrum of illnesses in humans, ranging from asymptomatic infection or mild dengue fever (DF) to life-threatening dengue hemorrhagic fever (DHF) and dengue shock syndrome (DSS). Dengue is caused by four DENV serotypes; however, dengue pathogenesis is complex and poorly understood. Establishing a useful animal model that can exhibit dengue-fever-like signs similar to those in humans is essential to improve our understanding of the host response and pathogenesis of DENV. Although several animal models, including mouse models, non-human primate models, and a recently reported tree shrew model, have been investigated for DENV infection, animal models with clinical signs that are similar to those of DF in humans have not yet been established. Although animal models are essential for understanding the pathogenesis of DENV infection and for drug and vaccine development, each animal model has its own strengths and limitations. Therefore, in this review, we provide a recent overview of animal models for DENV infection and pathogenesis, focusing on studies of the antibody-dependent enhancement (ADE) effect in animal models.

## Introduction

Dengue, caused by dengue virus (DENV), is one of the most important arthropod-borne human viral infections in many tropical and subtropical areas [39, 128]. The geographic range of dengue is increasing due to several factors, including climate change, unplanned rapid urbanization and construction, high population densities, and ineffective vector-control strategies [37, 71]. A recent study highlighted the possibility of an enzootic cycle of DNEV maintenance and reported dengue positivity in a range of animals and birds, including pigs (34.1%), non-human primates (27.3%), marsupials (13%), birds (11%), bats (10.1%), horses (5.1%), bovids (4.1%), rodents (3.5%), dogs (1.6%), and other small animals (7.3%) [41]. However, their role as potential reservoirs for dengue transmission remains to be confirmed [41]. The mosquito *Aedes aegypti* and, to a lesser extent, *Aedes albopictus* are the primary vectors for DENV transmission [66]. One-third of the global population is at risk of DENV infection, with approximately 390 million infections occurring each year [11]. DENV is a positive-sense, single-stranded RNA virus belonging to the family *Flaviviridae* and the genus *Flavivirus* with a genome of 10.7 kb [62]. Other pathogenic viruses, including yellow fever virus (YFV), Japanese encephalitis virus (JEV), tick-borne encephalitis virus (TBEV), Usutu virus (USUV), West Nile virus (WNV), and Zika virus (ZIKV), also belong to the family *Flaviviridae* [90]. The DENV genome encodes three structural proteins – the capsid (C), membrane (M), and envelope (E) proteins – and seven nonstructural proteins, NS1, NS2A, NS2B, NS3, NS4A, NS4B, and NS5 [39, 62]. There are four genetically and antigenically distinct DENV serotypes: DENV-1, DENV-2, DENV-3, and DENV-4 [110]. Infection with one serotype may provide lifelong immunity against infection with a homologous DENV strain; however, only short-term immunity is maintained against heterologous infections [1]. Notably, a heterologous infection may develop into severe dengue, possibly due to antibody-dependent enhancement (ADE) [57]. DENV causes a spectrum of illness in humans, ranging from asymptomatic to mild dengue fever, as well as potentially life-threatening severe dengue, including dengue hemorrhagic fever (DHF) and dengue shock syndrome (DSS) [47]. Severe dengue is characterized by plasma leakage, hemorrhagic tendencies, organ failure, shock, and occasionally death [113]. Notably, DENV, in addition to being a human/mammalian virus, is able to infect mosquito midgut cells and other tissues and spread to the salivary glands, and infected mosquitos can transmit DENV to humans during feeding [40]. Moreover, it has been reported that mosquito-derived factors, including mosquito salivary gland extract, can exacerbate dengue pathogenesis [107], which indicates the importance of studying DENV in invertebrate hosts. In addition, the antiviral response of the mosquito during DENV replication might provide an important platform for developing alternative strategies for dengue control [25]. However, animal models are also invaluable tools for characterizing human viral infections and pathogenesis, and a suitable animal model is imperative for improving our understanding of the pathogenesis of dengue. To date, several animal models have been used in studies on dengue pathogenesis. However, many challenges remain to be overcome [22]. Moreover, animal models play a crucial role in the preclinical testing of antiviral drugs and vaccines. In this review, we provide a recent overview of mammalian models used in studies on DENV infection and pathogenesis. Our focus extends to the ADE phenomenon observed in animal models of secondary heterologous DENV infections.

## Animal models for DENV infection

**Fig. 1:**
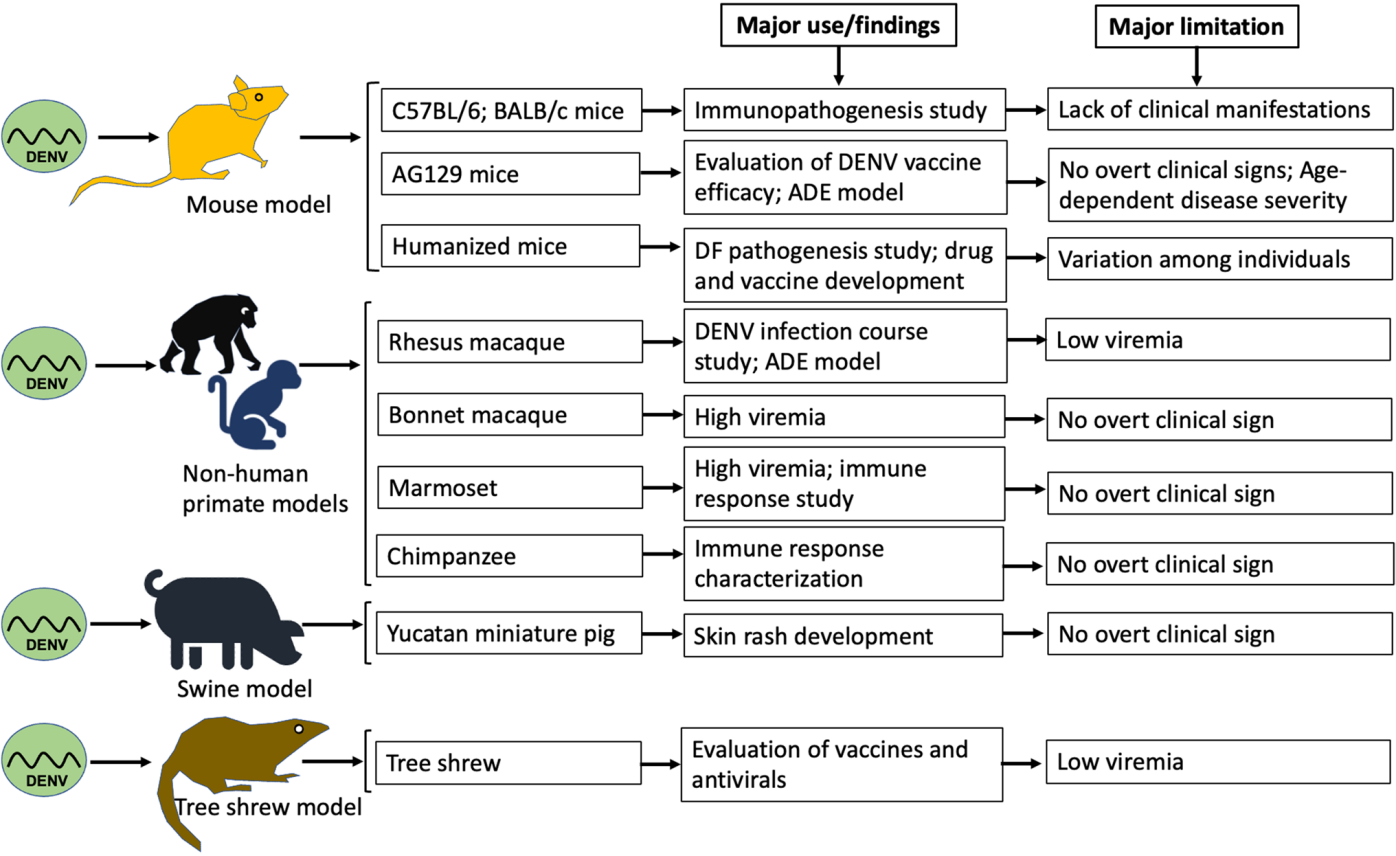
An overview of animal models used in DENV infection, including the major uses and limitations of each model

Animal models are invaluable tools for deciphering viral pathogenesis, allowing questions to be addressed that cannot be addressed using human trials. The establishment of a small-animal model is of great importance for the study of dengue, including research on host-virus interactions, disease pathogenesis, and antiviral and vaccine development. The immunopathogenesis of dengue is poorly understood, partly due to the lack of a suitable animal model of DENV infection. A suitable small-animal model whose infection manifestations resemble those of human dengue infection is critical, and the development of such a model has been long sought and challenging. In this context, several animal models have been investigated in DENV infection and pathogenesis studies [139], including mouse models, non-human primate (NHP) models, swine models, and a recently reported tree shrew model, as discussed in the sections below. A summary of DENV infections in animal models is shown in Table 1. In addition, we provide a graphical presentation (Fig. 1) of animal models used in DENV infection studies, highlighting the major uses and limitations of each model.

**Table 1:** Summary of DENV infection in animal models

Animal model	Immune status	DENV serotypes	Inoculation routes	Inoculation dose	Strengths	Limitations	References
C57BL/6 and BALB/c mice	Immunocompetent	DENV-2, DENV-3	IP, IV	Variable (1 × 10^4^ – 1 × 10^5^ PFU; 10^4^ TCID_50_	Useful in the study of immunopathogenesis	Very low viremia; no clinical manifestations	[27, 87, 129, 130]
AG129 mice	Immunodeficient	DENV-1 to DENV-4	SC, IP	Variable (1 × 10^3^ – 1 × 10^7^ PFU)	Allows for infection by all four DENV serotypes; allows antibody-mediated protection; production of ADE phenomenon; immunogenicity and protection testing	No overt clinical manifestations; limited immune response may not reflect natural infections; age-dependent disease severity	[7, 14, 74, 84, 102, 103, 127, 134]
Humanized mice	Immunodeficient	DENV-2	SC, ID, IP, IV, mosquito	Variable (1 × 10^4.7^ – 1 × 10^8^ PFU)	Clinical manifestations develop (viremia; thrombocytopenia occurs); suitable for the study of the pathogenesis of dengue fever; potentially useful for drug and vaccine development	Mouse-to-mouse variations; limited immune response	[3, 9, 26, 28, 52, 79, 80]
Rhesus monkey	Immunocompetent	DENV-1 to -4	SC	Variable (1 × 10^3.7^ – 1 × 10^7^ PFU)	Sustains viral replication; course of infection resembles DENV infection in humans; production of immune response and vaccine efficacy testing; ADE effect	High costs; low viremia; does not develop vascular leaks, DHF, or DSS	[12, 30, 35, 43, 69, 72]
Marmoset	Immunocompetent	DENV-1 to -4	SC	Variable (1.8 × 10^3^ – 6.7 × 10^7^ PFU)	High plasma viral load; cellular and humoral immune response	No overt clinical signs	[76, 85, 131]
Bonnet macaque	Immunocompetent	DENV-4	IV	1 × 10^6^ PFU	High viremia (2.2–4.0 × 10^6^ copies/mL); antibody response	No clinical symptoms	[56]
Chimpanzee	Immunocompetent	DENV-1 to -4	SC, ID	10^3^-10^6^ PFU	Detectable viremia; immune response (nAb production)	No overt clinical signs	[73, 106]
Swine (Yucatan miniature pig)	Immunocompetent	DENV-1	SC, IV	1 × 10^5^ or 1 × 10^7^ PFU	Viremia; Ab production; skin rash (IV inoculation)	No overt clinical signs	[21, 98]
Tree shrew	Immunocompetent	DENV-2, DENV-3	IV, SC	1.5 × 10^3^ PFU	Induces rise of body temperature, modest thrombocytopenia; may be suitable for the evaluation of antivirals and vaccines	Very low viremia; no manifestations of severe dengue	[53]

PFU, plaque-forming units; TCID_50_, median tissue culture infectious dose; IV, intravenous; SC, subcutaneous; ID, intradermal; IP, intraperitoneal

## Mouse models

Mouse models are ideal for investigating host–virus interactions due to the availability of immunological tools; however, wild-type mice lack susceptibility to DENV infection [129]. To date, an ideal mouse model for use in DENV infection and pathogenesis studies has yet to be established. Although immunocompetent mice are not readily infected by DENV, mouse models have been used frequently in DENV infection studies as a useful complement to *in vitro* and epidemiological data [136]. This is due in part to the fact that the conditions for infection of mice are well defined, and experimental parameters can be altered with relative ease. However, data generated using DENV-infected mice should be extrapolated to dengue in humans with caution due to the inherent limitations of animal models. Notably, a recent study demonstrated phenotypic differences between DENV in plasma and DENV that had undergone a single passage in a laboratory cell line, showing that the virus in plasma was 50 to 700 times more infectious than cell-culture-grown DENV, and this was attributed to the mature state of the plasma virions [94]. In an early study, Sabin and Schlesinger showed that DENV could be propagated in mice by intracerebral inoculation [99]. However, the initial adaptation of DENV to mice was tedious and required several consecutive passages. Furthermore, the virus propagated in mice could reproduce dengue in humans but was unable to infect cotton rats, hamsters, guinea pigs, or rabbits [99]. A more recent study showed that intraperitoneal (IP) inoculation with non-mouse-adapted DENV-2 strains resulted in successful infection of adult BALB/c mice, which had very low viremia without any clinical signs, except for increased serum ALT and AST levels. Histopathological analysis also revealed liver injury and the presence of viral antigens [87]. However, the level of DENV replication in wild-type mice is very low compared to that in humans [130]. Furthermore, it has been reported that intradermal inoculation of DENV-2 (strain 16681) induces systemic hemorrhage in immunocompetent C57BL/6 mice, with a high viral titer, macrophage infiltration, and tumor necrosis factor alpha (TNF-α) production [23]. A recent study showed that C57BL/6 mice sequentially infected with non-mouse-adapted DENV-1 (strain Puerto Rico/94) and DENV-2 (strain Tonga/74) had a low platelet count, internal hemorrhages, and increased liver enzymes in serum [119]. Moreover, the transfer of DENV-1-specific CD8^+^T lymphocytes before DENV-2 challenge has been found to enhance disease severity [119], suggesting that heterotypic anti-DENV CD8^+^ T lymphocytes play a role in the manifestation of severe dengue disease [114]. This will need to be investigated further in future studies. Infection of BALB/c mice with different DENV serotypes has also been studied [16, 50, 100], and a previous study showed that infection of adult BALB/c mice with mouse-adapted DENV-3 resulted in lethality, the rate of which increased with increasing inoculum, with increased IFN-γ expression preceded by increased IL-12 and IL-18 expression [27]. Moreover, severe disease manifestations with increased lethality were observed in IFN-γ(-/-) mice, suggesting that IFN-γ plays a role in resistance to DENV infection [27]. It has also been shown that IP inoculation of C57BL/6 mice with DENV-1 results in viremia, and produces signs of dengue, such as thrombocytopenia, spleen hemorrhage, liver damage, and increased production of IFN-γ and TNF-α [34]. Recently, experiments with 3-day-old suckling C57BL/6 and BALB/c mice infected with the DENV-2 New Guinea C (NGC) strain demonstrated that C57BL/6 pups are more susceptible than BALB/c pups and exhibit elevated liver enzymes and extended viremia [16]. Moreover, DENV-infected C57BL/6 pups showed a marked Th1 bias compared to BALB/c mice [16].

A previous study showed that DENV-2 infection in AG129 mice (lacking type I and II IFN receptors) results in paralysis [54], a condition of the central nervous system that is not commonly observed in cases of dengue in humans; however, the number of reports of neurological symptoms in dengue infections is increasing [68]. Although a mouse model of neuroinfection was established previously [122], a mouse-adapted DENV-2 strain, D2S10, has been shown to induce human DHF/DSS-like disease in AG129 mice without neurologic abnormalities [109, 134]. Moreover, DENV-2 (strain D2S10) was found to be more virulent than its parental strain (DENV-2 PL046, Taiwanese isolate; *Aedes albopictus* C6/36 cell culture adapted), producing a lethal but non-paralytic disease, with increased vascular permeability [109]. A non-canonical IRF-3-, IRF-5-, and IRF-7-independent antiviral defense mechanism against severe dengue, mediated by IRF-1, has been demonstrated in AG129 mice [18], suggesting that IRF-1 plays a role in DENV infection. DENV susceptibility has been demonstrated in mice lacking STAT1, STAT2, and type 1 IFN receptors [89, 108]. DENV infection in Cardif-deficient mice showed increased DENV RNA in serum and lymphoid tissues compared to wild-type mice, suggesting a role of Cardif in DENV infection [88]. A previous study demonstrated that conditional IFNAR mice (mice lacking the IFN receptor on either CD11c- or LysM-expressing cells) can induce a better immune response in DENV infection than IFNAR^−/−^ mice and may be useful for screening future vaccine candidates [140].

Clinical isolates of DENV-1 and DENV-2 have been reported to induce severe dengue in AG129 mice, which are suitable for drug testing and dengue pathogenesis studies [124]. However, the infectivity of DENV in mice may differ among virus strains. A recent study demonstrated that severe dengue manifested with vascular leakage in type I and type II IFN receptor double knockout C57BL/6 mice (AG6) [138]. AG129 mice have the potential to be used in studies evaluating the immunogenicity and efficacy of dengue vaccine candidates [14]. AG129 mice are mainly used as a lethal DENV infection model. IP inoculation of AG129 mice with DENV-4 (strain TVP-376) (10^7^ PFU) was found to result in high viral loads in multiple tissues, with the mice succumbing 5 days after infection [102]. Another study showed that IP inoculation of AG129 mice with non-mouse-adapted DENV-3 produced high viral loads and histological changes in tissues, including the spleen and liver, and increased cytokine levels in serum [103]. Moreover, an ADE-independent lethal disease has been demonstrated after a high-titer IP challenge with non-mouse-adapted DENV-1 (strain West Pacific 74) in AG129 mice [74]. Notably, a recent study showed a milder form of dengue in AG129 mice infected with a low-passage, non-mouse-lethal strain, DENV-3 D83-144 [104], suggesting that the AG129 mouse model could be used to investigate both mild and severe forms of the disease.

Severe combined immunodeficiency (SCID) mice lack functional T and B cells. Therefore, they lack both humoral and cellular responses and can accept engraftment of human cells, making them susceptible to DENV infection [26, 70]. However, humanized mice are expensive and laborious to prepare and frequently exhibit mouse-to-mouse variation, limiting their application in vaccine development studies [3]. Despite their limited immune response, humanized chimeric mice have been used as relevant models for studying dengue fever pathogenesis in humans, producing fever, viremia, erythema, and thrombocytopenia; however, severe disease has yet been reported in these mice [9, 28, 79, 80]. Humanized chimeric mice may also offer the possibility of studying the human immune response *in vivo*. A previous study established non-obese diabetic (NOD)/SCID interleukin-2 receptor gamma-chain knockout (NOD-SCID IL2rγ(null)) mice engrafted with human hematopoietic stem cells for DENV infection and showed functional DENV-specific T cell responses [51]. These mice could be used to investigate the role of cross-reactive T cells in DENV infections, including whether they are protective or pathogenic upon subsequent infection [31]. Another study established humanized BLT-NSG mice (NOD-SCID IL2rγ(null) mice co-transplanted with human fetal thymus and liver tissues) and demonstrated their suitability for evaluating the immunogenicity of candidate dengue vaccines [52]. Notably, DENV infection has also been studied in immunocompetent humanized RAG2(-/-)gamma(c)(-/-) (RAG-hu) mice. In that study, anti-DENV antibodies were reported in 10 of 16 RAG-hu mice, and three of them had neutralizing antibodies (nAbs) [63]. Overall, although studies on DENV infection in mouse models have allowed the identification of key viral and host factors, further investigations in both immunodeficient and immunocompetent mouse models may help to improve our understanding of host-virus interactions [36], which will aid in the development of antivirals and vaccines.

## Non-human primate (NHP) models

NHPs provide more advantages as an animal model of human dengue due to a close physiological and genetic relationship between humans and NHPs [32]. Various NHP models of DENV infection, including rhesus macaques (*Macaca mulatta*), cynomolgus macaques (*Macaca fascicularis*), green monkeys (*Chlorocebus sabaeus*), owl monkeys (*Aotus nancymaae*), chimpanzees (*Pan troglodytes*), spider monkeys (*Ateles geoffroyi*), pig-tailed macaques (*Macaca nemestrina*), common marmosets (*Callithrix jacchus*), patas monkeys (*Erythrocebus patas*), squirrel monkeys (*Saimiri sciureus*), and white-handed gibbons (*Hylobates lar*), have been investigated, as reviewed previously [4]. Recently, bonnet macaques (*Macaca radiata*) have also been used in experimental studies of DENV infection [56]. Studies using WNV demonstrated that mosquitoes inoculate 10^4^ to 10^6^ PFU of WNV per bite [117], which is also assumed for DENV. Therefore, infection of animals with 10^4^–10^6^ PFU of DENV is considered to mimic the inoculum in a mosquito bite. The viremia levels and antibody responses during primary and secondary DENV infections in NHP models have been reviewed recently [81]. Here, we provide an overview of NHP models that are frequently used to enhance our understanding of DENV infection and pathogenesis.

### Rhesus macaque model

The rhesus macaque is the first model that was used in the investigation of dengue etiology via the inoculation of defibrinated blood obtained from dengue patients [67]. Rhesus macaques are susceptible to DENV infection and exhibit detectable viremia and immune responses, including T-cell responses, but they do not develop overt clinical signs of infection [19, 42, 43, 48, 49, 125]. However, some rhesus macaques show low platelet counts after subcutaneous DENV infection [43, 44]. According to an estimate from a previous study, the time to viremia in rhesus macaques varies from 2.63 to 5.13 days, depending on the serotype [4], suggesting that the DENV serotype plays a role in the pathogenesis of dengue. Although rhesus macaques have frequently been used in dengue vaccine evaluation studies [5, 10, 13, 30, 69, 95–97, 118], vaccine efficacy should be carefully confirmed to avoid overestimation of vaccine efficacy, since viremia is much lower in NHPs than in human dengue [8]. A previous study demonstrated that the passive transfer of a monoclonal antibody (mAb) (IgG 5H2 ΔD) specific for DENV-4 protected rhesus monkeys against DENV-4 infection [65]. Intravenous inoculation with a high dose (10^7^ PFU) of DENV-2 strain 16681 produced hemorrhage after 3–5 days of infection, with modest thrombocytopenia and neutropenia concomitant with a slight decrease in hemoglobin and hematocrit [86], suggesting that the inoculation route and dose may have an effect on dengue pathogenesis. Moreover, rhesus macaques could be used as an animal model for characterizing the early events in dengue virus infection to identify blood components that may be involved in pathogenesis [86].

### Cynomolgus macaques

Cynomolgus macaques have also been used in DENV infection and vaccine efficacy studies for decades [5, 15, 24, 38, 123]. Similar to other NHP models, no overt clinical signs of dengue have been reported in this animal; however, nAbs and infrequent viremia have been reported [5, 123]. A previous study reported cynomolgus macaques possessing dengue-specific IgM and IgG derived from natural infection [55], suggesting that cynomolgus macaques act as a reservoir for the virus, which may be an important characteristic for future investigations.

### Bonnet macaque model

Bonnet macaques are indigenous to southern India, where DENV circulates widely. These animals have been used to study experimental and natural infections caused by flaviviruses. A previous study demonstrated the presence of nAbs in bonnet macaques naturally infected with DENV [133]. Furthermore, a recent study demonstrated that intravenous inoculation of bonnet monkeys with DENV-4 resulted in high viremia without clinical symptoms [56]. However, DENV serotypes 1, 2, and 3 remain to be tested in this animal model.

### Marmoset model

Animal models that consistently develop high levels of viremia are essential for the development of protective and preventive measures against disease. The common marmoset (*Callithrix jacchus*) of the family Callitrichidae (body weight, approximately 350–400 g) is native to northeastern Brazil [20]. The marmoset has been a useful primary and secondary dengue infection model, consistently producing high levels of viremia in primary and secondary DENV infections [76, 85]. It has been demonstrated that all four DENV serotypes produce a high plasma viral load, with DENV-2 viral RNA (vRNA) detected in lymphoid (lymph nodes, spleen, thymus, and bone marrow) and non-lymphoid organs [85]. The development of protective antibodies has been demonstrated in marmosets [82], suggesting the potential of marmosets for evaluation of the immunogenicity of candidate dengue vaccines [85]. During secondary infection with heterotypic DENV in marmosets, high levels of viremia, IgM and IgG responses, and serotype cross-reactive nAb responses were produced, resembling secondary DENV infection in humans [76]. The dynamics of cellular immune responses in the acute phase of DENV infection in a marmoset model have been demonstrated, where the responses of CD4/CD8 central memory T and NKT cells were found to be greatly induced [131]. In another study, Moi et al. demonstrated pathological changes in the kidney during DENV infection in marmosets, as observed in some dengue patients, highlighting the potential of the marmoset model as a suitable model for dengue pathogenesis studies [75].

### Chimpanzee model

Chimpanzees are genetically very close to humans and have been used in previous studies as DENV infection models [48, 73, 106]. They have been found to experience detectable subclinical disease without any overt clinical signs of dengue [106]. However, more recently, the use of chimpanzees in experimental DENV infections has become difficult due to its high costs and close association with animal welfare issues.

## Swine model

Swine share physiological similarities with humans and have several advantageous features, including their low cost and the availability of immunological reagents, making them an efficient animal model. A strain of Yucatan miniature pig, *Sus scrofa*, was found to exhibit physiological and immunological responses similar to those in humans [21, 83]. These animals were also found to be susceptible to some viruses of the family *Flaviviridae*, including JEV, YFV, and Murray Valley encephalitis virus [126]. A novel Yucatan miniature swine model has previously been described in which subcutaneous (SC) infection with DENV-1 resulted in detectable viremia with production of nAbs against intravenous (IV) inoculation of DENV-1, and infection of DENV-1 produced nAbs without detectable viremia [21]. Moreover, skin rash and dermal edema developed after subsequent DENV-1 reinfection in pigs subcutaneously infected with DENV-1 [21]. Although preliminary data are promising, further studies are required to develop Yucatan miniature swine as a small-animal model for dengue pathogenesis studies. These studies need to examine the susceptibility of the animals to other DENV subtypes, the level and duration of viremia, and development of clinical signs of dengue.

## Tree shrew model

The tree shrew is a small mammal belonging to the family Tupaiidae and the genus *Tupaia* [121], and it has been listed in the Lower Risk Category (Subcategory: Least Concern) of the International Union for Conservation of Nature (IUCN) Category of Threat (source: https://www.portals.iucn.org/library/sites/library/files/documents/1995-059.pdf; accessed on August 22, 2021). The tree shrew is an emerging experimental animal model with a higher degree of genetic similarity to primates than to rodents [33, 101]. Previous studies have demonstrated that tree shrews are a promising animal model for several important viral infections in humans, including hepatitis B virus, hepatitis C virus, influenza virus, and ZIKV [59]. In an *in vitro* study, we showed the susceptibility of tupaia lung fibroblast cells to infection with all four DENV serotypes [58]. Moreover, we found that the expression of TLR8 and IL-8 mRNA was upregulated in DENV-infected tupaia cells compared to uninfected cells [58]. In addition, silencing of TLR8 enhanced viral replication in these cells, indicating an antiviral role of TLR8 in DENV-infected tupaia cells [58]. Notably, a recent study established a tree shrew model for ZIKV with notable dermatological manifestations of skin rashes in ZIKV-infected tree shrews, as is commonly observed in human patients [137]. Furthermore, Jiang et al. described a novel model of dengue in tree shrews in which IV or SC inoculation with DENV-2 and DENV-3 gave rise to disease signs with features of dengue in humans [53]. Notably, DENV-infected tree shrews showed a significant increase in body temperature compared to uninfected individuals. Although the proliferation of DENV and pathological changes in the brain have been reported, viremia, a major clinical manifestation of DENV infection, is very low [53]. Moreover, vascular leakage, the hallmark of severe dengue [139], has not yet been demonstrated in DENV-infected tree shrews. Thus, additional research is needed, including experimental infection with other DENV serotypes. Notably, research findings on this animal model have been limited by the individual variability of wild tree shrews, requiring further investigation of the use of inbred tree shrews, which should reduce individual variability. However, using tree shrews as an immunocompetent model, tree-shrew-adapted DENV strains could be developed to overcome the problem of low-level viremia and could be used in host-virus interaction studies, as well as for preclinical testing of candidate dengue vaccines and antivirals. Furthermore, the tree shrew model could be applied to investigate ADE effects in secondary heterologous DENV infections. Although tree shrews are a promising small-animal model for human viral infections, their extensive use is still hampered by the lack of availability of tree-shrew-specific reagents and an inbred line of tree shrews [60].

## Transcriptional response to DENV infection in different models

Transcriptional changes in response to DENV infection may vary among individuals, influencing the outcome of infection. A proper understanding of transcriptional changes in response to DENV infection is important for the development of targeted immunomodulatory strategies to treat and control DENV infection. Therefore, use of different models for studying the transcriptional response to DENV infection is important for understanding host-DENV interactions. DENV infection in immunocompetent mice results in metabolic dysregulation and inflammatory responses in the spleen and liver, with activation of NK, NKT, and CD8^+^ T cells, similar to that seen in human cases [78]. It has been reported that the transcriptional response of PBMCs, including an increase in the number of activated CD4^+^ T cells correlates with the control of DENV infection in asymptomatic cases [111]. DENV-infected children in Nicaragua were shown to exhibit changes in gene expression, including increased expression of genes associated with the mitotic cell cycle and B cell differentiation and decreased expression of genes associated with signal transduction and cell adhesion [92]. Several studies have shown an association between changes in cytokine profiles and disease severity [92, 93]. The characterization of the immunotranscriptome response in humans before, during, and after infection with a partially attenuated rDENV-2Δ30 virus revealed the induction of inflammatory genes, including type I IFN, during viremia, which returned to baseline after viral clearance [46]. However, immune regulation pathways, including myeloid, migratory, humoral, and growth factor pathways, were reported to be at non-baseline levels post-viremia [46]. A recent study showed that DENV infection in AG129 mice can cause changes in two important components of the alternative complement pathway: factor B (FB) and factor H (FH). An increase in FB levels and a decrease in FH levels were observed at 3 dpi [17]. However, like in humans, terminal disease was associated with a decrease in FB and FH [17]. Inoculation with a live attenuated tetravalent dengue vaccine based on a DENV-2 backbone induced significant early transcriptional changes in the peripheral blood of cynomolgus macaques, with induction of 595 gene transcripts, including type I IFN, on days 1, 3, 5, and 7, compared to baseline and placebo-treated animals [116]. The transcriptional changes in rhesus macaques after 5 days of DENV-1 infection revealed a strong induction of the innate antiviral immune response, including the production of myxoprotein, 2’,5’-oligoadenylate synthetase, phospholipid scramblase 1, and viperin [105]. In addition, upregulation of IFN regulatory element 7, ISG15, and protein ligases linked to the ISGylation process was observed [105]. As observed in humans, CD14^+^CD16^+^ monocyte counts in the blood and lymph nodes were boosted after DENV infection in rhesus macaques [64]. A transcriptional response study of *Aedes aegypti* with a variable extrinsic incubation period (EIP) for DENV revealed that mosquitoes with a short EIP show less-active immune responses with higher levels of protein translation and homeostasis of calcium ions and that mosquitoes with a longer EIP may show slower metabolism [61]. Studies of the transcriptional response to DENV infection in different models appear to be an important way to gain a better understanding of host-DENV interactions, which may suggest new control strategies for DENV infection.

## Effects of ADE in animal models of DENV infection

Antibody-dependent enhancement (ADE) of disease is a public health concern for the development of vaccines and antibody therapies, as the same mechanisms that underlie antibody protection against viruses may also have the potential to enhance infection or affect immunopathology negatively [6]. Vaccines that provide long-term protection against each of the four DENV serotypes by inducing nAbs are required for the control of dengue and to prevent severe dengue [82]. However, the development of vaccines against DENV is largely threatened by the ADE phenomenon, and previous DENV vaccine studies have revealed severe safety risks related to ADE, which has resulted in failed vaccine trials [29, 112]. There is a need to elucidate the detailed mechanism of ADE, and a suitable animal model is essential for this purpose. *In vivo* evidence of ADE during DENV infection was first described in rhesus macaques [45]. Injection of an anti-DENVmAb (IgG 1A5) into juvenile rhesus monkeys was reported to lead to a 3- to 100-fold increase in viremia by ADE [35]. A recent study further extended the use of the macaque model in the evaluation of ADE and demonstrated increased levels of viremia, aspartate transaminase (AST), IL-10, IL-18, and IFN-γ and decreased levels of IL-12 in vaccinated macaques compared to non-vaccinated macaques, suggesting that vaccination may trigger ADE in DENV infection [12]. In a previous study, the formation of infectious DENV–Ab immune complexes was observed in marmosets after intravenous administration of anti-DENV mAbs and infection with DENV; however, the overall enhancement of viremia by passively transferred antibody was limited [77].

The AG129 mouse model can also be used for studying the ADE phenomenon, and previous studies have shown that administration of DENV-specific antibodies to AG129 mice infected with mouse-adapted DENV-2 (strain D2S10) produced an effect resembling human DHF/DSS [7, 134]. Moreover, a recent study provided evidence of ADE in AG129 mice, showing that DENV-2 infection of young mice born to DENV-1-immune mothers caused early death, with increased viremia and vascular leakage compared to DENV-2-infected mice born to dengue-naïve mothers [84]. A previous study indicated that CD8^+^ T cell responses play a role in reducing Ab-mediated severe dengue disease in a mouse model [135]; however, this requires further investigation. Another study found that specific deletion of IFNAR in subsets of murine myeloid cells (LysM Cre(+) IFNAR(flox/flox) resulted in enhanced DENV replication *in vivo* [91]. Moreover, it has been demonstrated that DENV serotype 2 or 3 infection in LysM Cre(+) IFNAR(flox/flox) mice after the administration of subneutralizing cross-reactive anti-DENV mAbs resulted in ADE [91]. Therefore, balanced and durable immunity to all four DENV serotypes is needed for the development of a dengue vaccine to avoid the risk of ADE in subsequent infections. Thus, a suitable ADE animal model is needed to elucidate the mechanisms of ADE. To summarize, Table 2 shows a comparison of DENV infection characteristics in different animal models, highlighting the need for further research to develop a suitable small animal model for dengue resembling the disease in humans.

**Table 2:** Comparison of DENV infection characteristics in different animal models

**Characteristic**	**Animal model**
**Viral replication**	
High viremia	AG129 mice; humanized mice; marmoset; bonnet macaque
Low viremia	Rhesus macaque; chimpanzee; C57BL/6; BALB/c mice; tree shrew
**Clinical signs**	
Dengue clinical manifestations	Humanized mice; AG129 mice (DENV serotype and mice-age dependent)
Fever	Marmoset; humanized mice, tree shrew
Thrombocytopenia	Humanized mice; AG129 mice; marmoset; tree shrew
Leukopenia	Marmoset
Skin rash	Swine
Liver involvement	AG129 mice; C57BL/6 mice
Kidney involvement	C57BL/6 mice; BALB/c mice
Vascular leak syndrome	AG129 mice (DENV-2 infection)
Neurological signs (not commonly seen in human dengue)	AG129 mice
**Immune response**	
Seroconversion	Marmoset; bonnet macaque; chimpanzee; mice; tree shrew; swine
Production of neutralizing antibodies	Chimpanzee; rhesus monkey; cynomolgus monkey; marmoset; mice models
T cell immunity	Marmoset
Antibody-dependent enhancement	Rhesus macaque; LysM Cre(+) Ifnar(flox/flox) mice; AG129 mice

## Conclusions

Although substantial progress has been made in our understanding of dengue pathogenesis using different animal models of DENV infection, none of the existing animal models represents the ideal model for the study of the pathogenesis of dengue. Moreover, there are large variations in the host response to DENV infection in humans and animal models. For example, the DENV NS2B3 protease complex cleaves human STING, but not mouse STING [2, 115, 132]. Moreover, recent studies have shown that DENV cannot inactivate STING in most primates, including chimpanzees, rhesus macaques, and common marmosets [115]. Therefore, data obtained from the study of DENV infection and pathogenesis in animal models should be regarded with caution. Moreover, as the global burden of dengue continues to increase, combined with the lack of an effective pan-serotype dengue vaccine [120], which is important to avoid the threat of ADE, the lack of a suitable small animal model is likely to hinder the development of effective vaccines and treatments. Moreover, the factors involved in the mechanisms underlying ADE need to be confirmed in a suitable animal model. In this context, it is worth noting the recent development of an immunocompetent dengue tree shrew model [53], which requires further validation and whose use in the study of dengue pathogenesis and vaccine efficacy should be extended in the future.

## References

[1] Aguas R, Dorigatti I, Coudeville L, Luxemburger C, Ferguson NM (2019). Cross-serotype interactions and disease outcome prediction of dengue infections in Vietnam. Sci Rep.

[2] Aguirre S, Maestre AM, Pagni S, Patel JR, Savage T, Gutman D, Maringer K, Bernal-Rubio D, Shabman RS, Simon V, Rodriguez-Madoz JR, Mulder LC, Barber GN, Fernandez-Sesma A (2012). DENV inhibits type I IFN production in infected cells by cleaving human STING. PLoS Pathog.

[3] Akkina R, Berges BK, Palmer BE, Remling L, Neff CP, Kuruvilla J, Connick E, Folkvord J, Gagliardi K, Kassu A, Akkina SR (2011). Humanized Rag1−/− gammac−/− mice support multilineage hematopoiesis and are susceptible to HIV-1 infection via systemic and vaginal routes. PLoS ONE.

[4] Althouse BM, Durbin AP, Hanley KA, Halstead SB, Weaver SC, Cummings DA (2014). Viral kinetics of primary dengue virus infection in non-human primates: a systematic review and individual pooled analysis. Virology.

[5] Angsubhakorn S, Yoksan S, Bhamarapravati N, Moe JB, Marchette NJ, Pradermwong A, Sahaphong S (1988). Dengue-4 vaccine: neurovirulence, viraemia and immune responses in rhesus and cynomolgus monkeys. Trans R Soc Trop Med Hyg.

[6] Arvin AM, Fink K, Schmid MA, Cathcart A, Spreafico R, Havenar-Daughton C, Lanzavecchia A, Corti D, Virgin HW (2020). A perspective on potential antibody-dependent enhancement of SARS-CoV-2. Nature.

[7] Balsitis SJ, Williams KL, Lachica R, Flores D, Kyle JL, Mehlhop E, Johnson S, Diamond MS, Beatty PR, Harris E (2010). Lethal antibody enhancement of dengue disease in mice is prevented by Fc modification. PLoS Pathog.

[8] Barban V, Mantel N, De Montfort A, Pagnon A, Pradezynski F, Lang J, Boudet F (2018). Improvement of the dengue virus (DENV) nonhuman primate model via a reverse translational approach based on dengue vaccine clinical efficacy data against DENV-2 and -4. J Virol.

[9] Bente DA, Melkus MW, Garcia JV, Rico-Hesse R (2005). Dengue fever in humanized NOD/SCID mice. J Virol.

[10] Bernardo L, Izquierdo A, Alvarez M, Rosario D, Prado I, Lopez C, Martinez R, Castro J, Santana E, Hermida L, Guillen G, Guzman MG (2008). Immunogenicity and protective efficacy of a recombinant fusion protein containing the domain III of the dengue 1 envelope protein in non-human primates. Antiviral Res.

[11] Bhatt S, Gething PW, Brady OJ, Messina JP, Farlow AW, Moyes CL, Drake JM, Brownstein JS, Hoen AG, Sankoh O, Myers MF, George DB, Jaenisch T, Wint GR, Simmons CP, Scott TW, Farrar JJ, Hay SI (2013). The global distribution and burden of dengue. Nature.

[12] Borges MB, Marchevsky RS, Carvalho Pereira R, da Silva MY, Almeida Mendes LG, Diniz-Mendes L, Cruz MA, Tahmaoui O, Baudart S, Freire M, Homma A, Schneider-Ohrum K, Vaughn DW, Vanloubbeeck Y, Lorin C, Malice MP, Caride E, Warter L (2019). Detection of post-vaccination enhanced dengue virus infection in macaques: an improved model for early assessment of dengue vaccines. PLoS Pathog.

[13] Bray M, Men R, Lai CJ (1996). Monkeys immunized with intertypic chimeric dengue viruses are protected against wild-type virus challenge. J Virol.

[14] Brewoo JN, Kinney RM, Powell TD, Arguello JJ, Silengo SJ, Partidos CD, Huang CY, Stinchcomb DT, Osorio JE (2012). Immunogenicity and efficacy of chimeric dengue vaccine (DENVax) formulations in interferon-deficient AG129 mice. Vaccine.

[15] Butrapet S, Rabablert J, Angsubhakorn S, Wiriyarat W, Huang C, Kinney R, Punyim S, Bhamarapravati N (2002). Chimeric dengue type 2/type 1 viruses induce immune responses in cynomolgus monkeys. Southeast Asian J Trop Med Public Health.

[16] Byrne AB, Garcia AG, Brahamian JM, Mauri A, Ferretti A, Polack FP, Talarico LB (2021). A murine model of dengue virus infection in suckling C57BL/6 and BALB/c mice. Anim Model Exp Med.

[17] Cabezas-Falcon S, Norbury AJ, Hulme-Jones J, Klebe S, Adamson P, Rudd PA, Mahalingam S, Ong LC, Alonso S, Gordon DL, Carr JM (2021). Changes in complement alternative pathway components, factor B and factor H during dengue virus infection in the AG129 mouse. J Gen Virol.

[18] Carlin AF, Plummer EM, Vizcarra EA, Sheets N, Joo Y, Tang W, Day J, Greenbaum J, Glass CK, Diamond MS, Shresta S (2017). An IRF-3-, IRF-5-, and IRF-7-independent pathway of dengue viral resistance utilizes IRF-1 to stimulate type I and II interferon responses. Cell Rep.

[19] Carrington LB, Ponlawat A, Nitatsukprasert C, Khongtak P, Sunyakumthorn P, Ege CA, Im-Erbsin R, Chumpolkulwong K, Thaisomboonsuk B, Klungthong C, Yoon IK, Ellison D, Macareo L, Simmons CP (2020). Virological and immunological outcomes in rhesus monkeys after exposure to dengue virus-infected Aedes aegypti mosquitoes. Am J Trop Med Hyg.

[20] Carrion R, Patterson JL (2012). An animal model that reflects human disease: the common marmoset (Callithrix jacchus). Curr Opin Virol.

[21] Cassetti MC, Durbin A, Harris E, Rico-Hesse R, Roehrig J, Rothman A, Whitehead S, Natarajan R, Laughlin C (2010). Report of an NIAID workshop on dengue animal models. Vaccine.

[22] Chan KW, Watanabe S, Kavishna R, Alonso S, Vasudevan SG (2015). Animal models for studying dengue pathogenesis and therapy. Antiviral Res.

[23] Chen HC, Hofman FM, Kung JT, Lin YD, Wu-Hsieh BA (2007). Both virus and tumor necrosis factor alpha are critical for endothelium damage in a mouse model of dengue virus-induced hemorrhage. J Virol.

[24] Chen L, Ewing D, Subramanian H, Block K, Rayner J, Alterson KD, Sedegah M, Hayes C, Porter K, Raviprakash K (2007). A heterologous DNA prime-Venezuelan equine encephalitis virus replicon particle boost dengue vaccine regimen affords complete protection from virus challenge in cynomolgus macaques. J Virol.

[25] Chen T-Y, Lee Y, Wang X, Mathias D, Caragata EP, Smartt CT (2021). Profiling transcriptional response of dengue-2 virus infection in midgut tissue of Aedes aegypti. Front Trop Dis.

[26] Coronel-Ruiz C, Gutierrez-Barbosa H, Medina-Moreno S, Velandia-Romero ML, Chua JV, Castellanos JE, Zapata JC (2020). Humanized mice in dengue research: a comparison with other mouse models. Vaccines (Basel).

[27] Costa VV, Fagundes CT, Valadao DF, Cisalpino D, Dias AC, Silveira KD, Kangussu LM, Avila TV, Bonfim MR, Bonaventura D, Silva TA, Sousa LP, Rachid MA, Vieira LQ, Menezes GB, de Paula AM, Atrasheuskaya A, Ignatyev G, Teixeira MM, Souza DG (2012). A model of DENV-3 infection that recapitulates severe disease and highlights the importance of IFN-gamma in host resistance to infection. PLoS Negl Trop Dis.

[28] Cox J, Mota J, Sukupolvi-Petty S, Diamond MS, Rico-Hesse R (2012). Mosquito bite delivery of dengue virus enhances immunogenicity and pathogenesis in humanized mice. J Virol.

[29] Dejnirattisai W, Jumnainsong A, Onsirisakul N, Fitton P, Vasanawathana S, Limpitikul W, Puttikhunt C, Edwards C, Duangchinda T, Supasa S, Chawansuntati K, Malasit P, Mongkolsapaya J, Screaton G (2010). Cross-reacting antibodies enhance dengue virus infection in humans. Science.

[30] Durbin AP, Karron RA, Sun W, Vaughn DW, Reynolds MJ, Perreault JR, Thumar B, Men R, Lai CJ, Elkins WR, Chanock RM, Murphy BR, Whitehead SS (2001). Attenuation and immunogenicity in humans of a live dengue virus type-4 vaccine candidate with a 30 nucleotide deletion in its 3′-untranslated region. Am J Trop Med Hyg.

[31] Elong Ngono A, Shresta S (2019). Cross-reactive T cell immunity to dengue and Zika viruses: new insights into vaccine development. Front Immunol.

[32] Estes JD, Wong SW, Brenchley JM (2018). Nonhuman primate models of human viral infections. Nat Rev Immunol.

[33] Fan Y, Huang ZY, Cao CC, Chen CS, Chen YX, Fan DD, He J, Hou HL, Hu L, Hu XT, Jiang XT, Lai R, Lang YS, Liang B, Liao SG, Mu D, Ma YY, Niu YY, Sun XQ, Xia JQ, Xiao J, Xiong ZQ, Xu L, Yang L, Zhang Y, Zhao W, Zhao XD, Zheng YT, Zhou JM, Zhu YB, Zhang GJ, Wang J, Yao YG (2013). Genome of the Chinese tree shrew. Nat Commun.

[34] Goncalves D, de Queiroz Prado R, Almeida Xavier E, Cristina de Oliveira N, da Matta Guedes PM, da Silva JS, Moraes Figueiredo LT, Aquino VH (2012). Immunocompetent mice model for dengue virus infection [corrected]. Sci World J.

[35] Goncalvez AP, Engle RE, St Claire M, Purcell RH, Lai CJ (2007). Monoclonal antibody-mediated enhancement of dengue virus infection in vitro and in vivo and strategies for prevention. Proc Natl Acad Sci USA.

[36] Guabiraba R, Ryffel B (2014). Dengue virus infection: current concepts in immune mechanisms and lessons from murine models. Immunology.

[37] Gubler DJ (2011). Dengue, urbanization and globalization: the unholy trinity of the 21(st) century. Trop Med Health.

[38] Guzman MG, Rodriguez R, Rodriguez R, Hermida L, Alvarez M, Lazo L, Mune M, Rosario D, Valdes K, Vazquez S, Martinez R, Serrano T, Paez J, Espinosa R, Pumariega T, Guillen G (2003). Induction of neutralizing antibodies and partial protection from viral challenge in Macaca fascicularis immunized with recombinant dengue 4 virus envelope glycoprotein expressed in Pichia pastoris. Am J Trop Med Hyg.

[39] Guzman MG, Halstead SB, Artsob H, Buchy P, Farrar J, Gubler DJ, Hunsperger E, Kroeger A, Margolis HS, Martinez E, Nathan MB, Pelegrino JL, Simmons C, Yoksan S, Peeling RW (2010). Dengue: a continuing global threat. Nat Rev Microbiol.

[40] Guzman MG, Gubler DJ, Izquierdo A, Martinez E, Halstead SB (2016). Dengue infection. Nat Rev Dis Primers.

[41] Gwee SXW, St John AL, Gray GC, Pang J (2021). Animals as potential reservoirs for dengue transmission: a systematic review. One Health.

[42] Halstead SB, Casals J, Shotwell H, Palumbo N (1973). Studies on the immunization of monkeys against dengue. I. Protection derived from single and sequential virus infections. Am J Trop Med Hyg.

[43] Halstead SB, Shotwell H, Casals J (1973). Studies on the pathogenesis of dengue infection in monkeys. I. Clinical laboratory responses to primary infection. J Infect Dis.

[44] Halstead SB, Shotwell H, Casals J (1973). Studies on the pathogenesis of dengue infection in monkeys. II. Clinical laboratory responses to heterologous infection. J Infect Dis.

[45] Halstead SB (1979). In vivo enhancement of dengue virus infection in rhesus monkeys by passively transferred antibody. J Infect Dis.

[46] Hanley JP, Tu HA, Dragon JA, Dickson DM, Rio-Guerra RD, Tighe SW, Eckstrom KM, Selig N, Scarpino SV, Whitehead SS, Durbin AP, Pierce KK, Kirkpatrick BD, Rizzo DM, Frietze S, Diehl SA (2021). Immunotranscriptomic profiling the acute and clearance phases of a human challenge dengue virus serotype 2 infection model. Nat Commun.

[47] Harris E, Videa E, Perez L, Sandoval E, Tellez Y, Perez ML, Cuadra R, Rocha J, Idiaquez W, Alonso RE, Delgado MA, Campo LA, Acevedo F, Gonzalez A, Amador JJ, Balmaseda A (2000). Clinical, epidemiologic, and virologic features of dengue in the 1998 epidemic in Nicaragua. Am J Trop Med Hyg.

[48] Harrison VR, Eckels KH, Sagartz JW, Russell PK (1977). Virulence and immunogenicity of a temperature-sensitive dengue-2 virus in lower primates. Infect Immun.

[49] Hickey AC, Koster JA, Thalmann CM, Hardcastle K, Tio PH, Cardosa MJ, Bossart KN (2013). Serotype-specific host responses in rhesus macaques after primary dengue challenge. Am J Trop Med Hyg.

[50] Jacome FC, Teixeira AL, Coutinho DD, Costa AD, Caldas GC, Nunes MA, Barth OM, Barreto-Vieira DF (2019). Secondary dengue infection in immunocompetent murine model leads to heart tissue damage. Acta Virol.

[51] Jaiswal S, Pearson T, Friberg H, Shultz LD, Greiner DL, Rothman AL, Mathew A (2009). Dengue virus infection and virus-specific HLA-A2 restricted immune responses in humanized NOD-scid IL2rgammanull mice. PLoS ONE.

[52] Jaiswal S, Smith K, Ramirez A, Woda M, Pazoles P, Shultz LD, Greiner DL, Brehm MA, Mathew A (2015). Dengue virus infection induces broadly cross-reactive human IgM antibodies that recognize intact virions in humanized BLT-NSG mice. Exp Biol Med (Maywood).

[53] Jiang L, Lu C, Sun Q (2021). Tree shrew as a new animal model for the study of dengue virus. Front Immunol.

[54] Johnson AJ, Roehrig JT (1999). New mouse model for dengue virus vaccine testing. J Virol.

[55] Kato F, Ishida Y, Kawagishi T, Kobayashi T, Hishiki T, Miura T, Igarashi T (2013). Natural infection of cynomolgus monkeys with dengue virus occurs in epidemic cycles in the Philippines. J Gen Virol.

[56] Kato F, Ishida Y, Kawakami A, Takasaki T, Saijo M, Miura T, Hishiki T (2018). Evaluation of Macaca radiata as a non-human primate model of Dengue virus infection. Sci Rep.

[57] Katzelnick LC, Gresh L, Halloran ME, Mercado JC, Kuan G, Gordon A, Balmaseda A, Harris E (2017). Antibody-dependent enhancement of severe dengue disease in humans. Science.

[58] Kayesh MEH, Kitab B, Sanada T, Hayasaka D, Morita K, Kohara M, Tsukiyama-Kohara K (2017). Susceptibility and initial immune response of Tupaia belangeri cells to dengue virus infection. Infect Genet Evol.

[59] Kayesh MEH, Hashem MA, Kitab B, Tsukiyama-Kohara K (2019). Pathogenesis and immune response caused by vector-borne and other viral infections in a Tupaia model. Microorganisms.

[60] Kayesh MEH, Sanada T, Kohara M, Tsukiyama-Kohara K (2021). Tree shrew as an emerging small animal model for human viral infection: a recent overview. Viruses.

[61] Koh C, Allen SL, Herbert RI, McGraw EA, Chenoweth SF (2018). The transcriptional response of Aedes aegypti with variable extrinsic incubation periods for dengue virus. Genome Biol Evol.

[62] Kuhn RJ, Zhang W, Rossmann MG, Pletnev SV, Corver J, Lenches E, Jones CT, Mukhopadhyay S, Chipman PR, Strauss EG, Baker TS, Strauss JH (2002). Structure of dengue virus: implications for flavivirus organization, maturation, and fusion. Cell.

[63] Kuruvilla JG, Troyer RM, Devi S, Akkina R (2007). Dengue virus infection and immune response in humanized RAG2(−/−)gamma(c)(−/−) (RAG-hu) mice. Virology.

[64] Kwissa M, Nakaya HI, Onlamoon N, Wrammert J, Villinger F, Perng GC, Yoksan S, Pattanapanyasat K, Chokephaibulkit K, Ahmed R, Pulendran B (2014). Dengue virus infection induces expansion of a CD14(+)CD16(+) monocyte population that stimulates plasmablast differentiation. Cell Host Microbe.

[65] Lai CJ, Goncalvez AP, Men R, Wernly C, Donau O, Engle RE, Purcell RH (2007). Epitope determinants of a chimpanzee dengue virus type 4 (DENV-4)-neutralizing antibody and protection against DENV-4 challenge in mice and rhesus monkeys by passively transferred humanized antibody. J Virol.

[66] Lambrechts L, Scott TW, Gubler DJ (2010). Consequences of the expanding global distribution of Aedes albopictus for dengue virus transmission. PLoS Negl Trop Dis.

[67] Lavinder CH, Francis E (1914). The etiology of dengue. an attempt to produce the disease in the rhesus monkey by the inoculation of defibrinated blood. J Infect Dis.

[68] Li GH, Ning ZJ, Liu YM, Li XH (2017). Neurological manifestations of dengue infection. Front Cell Infect Microbiol.

[69] Li L, Meng W, Horton M, DiStefano DR, Thoryk EA, Pfaff JM, Wang Q, Salazar GT, Barnes T, Doranz BJ, Bett AJ, Casimiro DR, Vora KA, An Z, Zhang N (2019). Potent neutralizing antibodies elicited by dengue vaccine in rhesus macaque target diverse epitopes. PLoS Pathog.

[70] Lin YL, Liao CL, Chen LK, Yeh CT, Liu CI, Ma SH, Huang YY, Huang YL, Kao CL, King CC (1998). Study of dengue virus infection in SCID mice engrafted with human K562 cells. J Virol.

[71] Lindsay SW, Wilson A, Golding N, Scott TW, Takken W (2017). Improving the built environment in urban areas to control Aedes aegypti-borne diseases. Bull World Health Organ.

[72] Marchette NJ, Halstead SB, Falkler WA, Stenhouse A, Nash D (1973). Studies on the pathogenesis of dengue infection in monkeys. 3. Sequential distribution of virus in primary and heterologous infections. J Infect Dis.

[73] Men R, Yamashiro T, Goncalvez AP, Wernly C, Schofield DJ, Emerson SU, Purcell RH, Lai CJ (2004). Identification of chimpanzee Fab fragments by repertoire cloning and production of a full-length humanized immunoglobulin G1 antibody that is highly efficient for neutralization of dengue type 4 virus. J Virol.

[74] Milligan GN, Sarathy VV, White MM, Greenberg MB, Campbell GA, Pyles RB, Barrett ADT, Bourne N (2017). A lethal model of disseminated dengue virus type 1 infection in AG129 mice. J Gen Virol.

[75] Moi ML, Omatsu T, Hirayama T, Nakamura S, Katakai Y, Yoshida T, Saito A, Tajima S, Ito M, Takasaki T, Akari H, Kurane I (2013). Presence of viral genome in urine and development of hematuria and pathological changes in kidneys in common marmoset (Callithrix jacchus) after inoculation with dengue virus. Pathogens.

[76] Moi ML, Takasaki T, Omatsu T, Nakamura S, Katakai Y, Ami Y, Suzaki Y, Saijo M, Akari H, Kurane I (2014). Demonstration of marmosets (Callithrix jacchus) as a non-human primate model for secondary dengue virus infection: high levels of viraemia and serotype cross-reactive antibody responses consistent with secondary infection of humans. J Gen Virol.

[77] Moi ML, Ami Y, Shirai K, Lim CK, Suzaki Y, Saito Y, Kitaura K, Saijo M, Suzuki R, Kurane I, Takasaki T (2015). Formation of infectious dengue virus-antibody immune complex in vivo in marmosets (Callithrix jacchus) after passive transfer of anti-dengue virus monoclonal antibodies and infection with dengue virus. Am J Trop Med Hyg.

[78] Morrison J, Rathore APS, Mantri CK, Aman SAB, Nishida A, St John AL (2017). Transcriptional profiling confirms the therapeutic effects of mast cell stabilization in a dengue disease model. J Virol.

[79] Mota J, Rico-Hesse R (2009). Humanized mice show clinical signs of dengue fever according to infecting virus genotype. J Virol.

[80] Mota J, Rico-Hesse R (2011). Dengue virus tropism in humanized mice recapitulates human dengue fever. PLoS ONE.

[81] Muhammad Azami NA, Takasaki T, Kurane I, Moi ML (2020). Non-human primate models of dengue virus infection: a comparison of viremia levels and antibody responses during primary and secondary infection among old world and new world monkeys. Pathogens.

[82] Murphy BR, Whitehead SS (2011). Immune response to dengue virus and prospects for a vaccine. Annu Rev Immunol.

[83] Na W, Yeom M, Choi IK, Yook H, Song D (2017). Animal models for dengue vaccine development and testing. Clin Exp Vaccine Res.

[84] Ng JK, Zhang SL, Tan HC, Yan B, Martinez JM, Tan WY, Lam JH, Tan GK, Ooi EE, Alonso S (2014). First experimental in vivo model of enhanced dengue disease severity through maternally acquired heterotypic dengue antibodies. PLoS Pathog.

[85] Omatsu T, Moi ML, Hirayama T, Takasaki T, Nakamura S, Tajima S, Ito M, Yoshida T, Saito A, Katakai Y, Akari H, Kurane I (2011). Common marmoset (Callithrix jacchus) as a primate model of dengue virus infection: development of high levels of viraemia and demonstration of protective immunity. J Gen Virol.

[86] Onlamoon N, Noisakran S, Hsiao HM, Duncan A, Villinger F, Ansari AA, Perng GC (2010). Dengue virus-induced hemorrhage in a nonhuman primate model. Blood.

[87] Paes MV, Pinhao AT, Barreto DF, Costa SM, Oliveira MP, Nogueira AC, Takiya CM, Farias-Filho JC, Schatzmayr HG, Alves AM, Barth OM (2005). Liver injury and viremia in mice infected with dengue-2 virus. Virology.

[88] Perry ST, Prestwood TR, Lada SM, Benedict CA, Shresta S (2009). Cardif-mediated signaling controls the initial innate response to dengue virus in vivo. J Virol.

[89] Perry ST, Buck MD, Lada SM, Schindler C, Shresta S (2011). STAT2 mediates innate immunity to Dengue virus in the absence of STAT1 via the type I interferon receptor. PLoS Pathog.

[90] Pierson TC, Diamond MS (2020). The continued threat of emerging flaviviruses. Nat Microbiol.

[91] Pinto AK, Brien JD, Lam CY, Johnson S, Chiang C, Hiscott J, Sarathy VV, Barrett AD, Shresta S, Diamond MS (2015). Defining new therapeutics using a more immunocompetent mouse model of antibody-enhanced dengue virus infection. MBio.

[92] Popper SJ, Gordon A, Liu M, Balmaseda A, Harris E, Relman DA (2012). Temporal dynamics of the transcriptional response to dengue virus infection in Nicaraguan children. PLoS Negl Trop Dis.

[93] Puc I, Ho TC, Yen KL, Vats A, Tsai JJ, Chen PL, Chien YW, Lo YC, Perng GC (2021). Cytokine signature of dengue patients at different severity of the disease. Int J Mol Sci.

[94] Raut R, Corbett KS, Tennekoon RN, Premawansa S, Wijewickrama A, Premawansa G, Mieczkowski P, Ruckert C, Ebel GD, De Silva AD, de Silva AM (2019). Dengue type 1 viruses circulating in humans are highly infectious and poorly neutralized by human antibodies. Proc Natl Acad Sci USA.

[95] Raviprakash K, Apt D, Brinkman A, Skinner C, Yang S, Dawes G, Ewing D, Wu SJ, Bass S, Punnonen J, Porter K (2006). A chimeric tetravalent dengue DNA vaccine elicits neutralizing antibody to all four virus serotypes in rhesus macaques. Virology.

[96] Raviprakash K, Wang D, Ewing D, Holman DH, Block K, Woraratanadharm J, Chen L, Hayes C, Dong JY, Porter K (2008). A tetravalent dengue vaccine based on a complex adenovirus vector provides significant protection in rhesus monkeys against all four serotypes of dengue virus. J Virol.

[97] Robert Putnak J, Coller BA, Voss G, Vaughn DW, Clements D, Peters I, Bignami G, Houng HS, Chen RC, Barvir DA, Seriwatana J, Cayphas S, Garcon N, Gheysen D, Kanesa-Thasan N, McDonell M, Humphreys T, Eckels KH, Prieels JP, Innis BL (2005). An evaluation of dengue type-2 inactivated, recombinant subunit, and live-attenuated vaccine candidates in the rhesus macaque model. Vaccine.

[98] Robinson JS (2005). Yucatan miniature swine as an animal model for dengue-1 disease.

[99] Sabin AB, Schlesinger RW (1945). Production of immunity to dengue with virus modified by propagation in mice. Science.

[100] Sakinah S, Priya SP, Kumari S, Amira F, Poorani K, Alsaeedy H, Ling MP, Chee HY, Higuchi A, Alarfaj AA, Munusamy MA, Murugan K, Taib CN, Arulselvan P, Rajan M, Neela VK, Hamat RA, Benelli G, Kumar SS (2017). Impact of dengue virus (serotype DENV-2) infection on liver of BALB/c mice: a histopathological analysis. Tissue Cell.

[101] Sanada T, Tsukiyama-Kohara K, Shin IT, Yamamoto N, Kayesh MEH, Yamane D, Takano JI, Shiogama Y, Yasutomi Y, Ikeo K, Gojobori T, Mizokami M, Kohara M (2019). Construction of complete *Tupaia belangeri* transcriptome database by whole-genome and comprehensive RNA sequencing. Sci Rep.

[102] Sarathy VV, Infante E, Li L, Campbell GA, Wang T, Paessler S, Robert Beatty P, Harris E, Milligan GN, Bourne N, Barrett ADT (2015). Characterization of lethal dengue virus type 4 (DENV-4) TVP-376 infection in mice lacking both IFN-alpha/beta and IFN-gamma receptors (AG129) and comparison with the DENV-2 AG129 mouse model. J Gen Virol.

[103] Sarathy VV, White M, Li L, Gorder SR, Pyles RB, Campbell GA, Milligan GN, Bourne N, Barrett AD (2015). A lethal murine infection model for dengue virus 3 in AG129 mice deficient in type I and II interferon receptors leads to systemic disease. J Virol.

[104] Sarathy VV, White M, Li L, Kaiser JA, Campbell GA, Milligan GN, Bourne N, Barrett ADT (2018). Characterization of a murine model of non-lethal, symptomatic dengue virus infection. Sci Rep.

[105] Sariol CA, Munoz-Jordan JL, Abel K, Rosado LC, Pantoja P, Giavedoni L, Rodriguez IV, White LJ, Martinez M, Arana T, Kraiselburd EN (2007). Transcriptional activation of interferon-stimulated genes but not of cytokine genes after primary infection of rhesus macaques with dengue virus type 1. Clin Vaccine Immunol.

[106] Scherer WF, Russell PK, Rosen L, Casals J, Dickerman RW (1978). Experimental infection of chimpanzees with dengue viruses. Am J Trop Med Hyg.

[107] Schmid MA, Glasner DR, Shah S, Michlmayr D, Kramer LD, Harris E (2016). Mosquito saliva increases endothelial permeability in the skin, immune cell migration, and dengue pathogenesis during antibody-dependent enhancement. PLoS Pathog.

[108] Shresta S, Sharar KL, Prigozhin DM, Snider HM, Beatty PR, Harris E (2005). Critical roles for both STAT1-dependent and STAT1-independent pathways in the control of primary dengue virus infection in mice. J Immunol.

[109] Shresta S, Sharar KL, Prigozhin DM, Beatty PR, Harris E (2006). Murine model for dengue virus-induced lethal disease with increased vascular permeability. J Virol.

[110] Simmons CP, Farrar JJ, Nguyen VV, Wills B (2012). Dengue. N Engl J Med.

[111] Simon-Loriere E, Duong V, Tawfik A, Ung S, Ly S, Casademont I, Prot M, Courtejoie N, Bleakley K, Buchy P, Tarantola A, Dussart P, Cantaert T, Sakuntabhai A (2017) Increased adaptive immune responses and proper feedback regulation protect against clinical dengue. Sci Transl Med 9:eaal5088 10.1126/scitranslmed.aal5088 28855396

[112] Sridhar S, Luedtke A, Langevin E, Zhu M, Bonaparte M, Machabert T, Savarino S, Zambrano B, Moureau A, Khromava A, Moodie Z, Westling T, Mascarenas C, Frago C, Cortes M, Chansinghakul D, Noriega F, Bouckenooghe A, Chen J, Ng SP, Gilbert PB, Gurunathan S, DiazGranados CA (2018). Effect of dengue serostatus on dengue vaccine safety and efficacy. N Engl J Med.

[113] Srikiatkhachorn A (2009). Plasma leakage in dengue haemorrhagic fever. Thromb Haemost.

[114] St John AL, Rathore APS (2019). Adaptive immune responses to primary and secondary dengue virus infections. Nat Rev Immunol.

[115] Stabell AC, Meyerson NR, Gullberg RC, Gilchrist AR, Webb KJ, Old WM, Perera R, Sawyer SL (2018). Dengue viruses cleave STING in humans but not in nonhuman primates, their presumed natural reservoir. Elife.

[116] Strouts FR, Popper SJ, Partidos CD, Stinchcomb DT, Osorio JE, Relman DA (2016). Early Transcriptional Signatures of the Immune Response to a Live Attenuated Tetravalent Dengue Vaccine Candidate in Non-human Primates. PLoS Negl Trop Dis.

[117] Styer LM, Kent KA, Albright RG, Bennett CJ, Kramer LD, Bernard KA (2007). Mosquitoes inoculate high doses of West Nile virus as they probe and feed on live hosts. PLoS Pathog.

[118] Sun W, Nisalak A, Gettayacamin M, Eckels KH, Putnak JR, Vaughn DW, Innis BL, Thomas SJ, Endy TP (2006). Protection of Rhesus monkeys against dengue virus challenge after tetravalent live attenuated dengue virus vaccination. J Infect Dis.

[119] Talarico LB, Batalle JP, Byrne AB, Brahamian JM, Ferretti A, Garcia AG, Mauri A, Simonetto C, Hijano DR, Lawrence A, Acosta PL, Caballero MT, Paredes Rojas Y, Ibanez LI, Melendi GA, Rey FA, Damonte EB, Harris E, Polack FP (2017). The role of heterotypic DENV-specific CD8(+)T lymphocytes in an immunocompetent mouse model of secondary dengue virus infection. EBioMedicine.

[120] Tremblay N, Freppel W, Sow AA, Chatel-Chaix L (2019). The interplay between dengue virus and the human innate immune system: a game of hide and seek. Vaccines (Basel).

[121] Tsukiyama-Kohara K, Kohara M (2014). Tupaia belangeri as an experimental animal model for viral infection. Exp Anim.

[122] Velandia-Romero ML, Acosta-Losada O, Castellanos JE (2012). In vivo infection by a neuroinvasive neurovirulent dengue virus. J Neurovirol.

[123] Velzing J, Groen J, Drouet MT, van Amerongen G, Copra C, Osterhaus AD, Deubel V (1999). Induction of protective immunity against Dengue virus type 2: comparison of candidate live attenuated and recombinant vaccines. Vaccine.

[124] Watanabe S, Chan KWK, Vasudevan SG (2016). Mouse model of dengue virus infection with serotypes 1 and 2 clinical isolates. Bio-Protoc.

[125] Watts DM, Burke DS, Harrison BA, Whitmire RE, Nisalak A (1987). Effect of temperature on the vector efficiency of Aedes aegypti for dengue 2 virus. Am J Trop Med Hyg.

[126] Williams DT, Daniels PW, Lunt RA, Wang LF, Newberry KM, Mackenzie JS (2001). Experimental infections of pigs with Japanese encephalitis virus and closely related Australian flaviviruses. Am J Trop Med Hyg.

[127] Williams KL, Zompi S, Beatty PR, Harris E (2009). A mouse model for studying dengue virus pathogenesis and immune response. Ann N Y Acad Sci.

[128] World Health Organization (2020) Dengue and severe dengue. https://www.who.int/news-room/fact-sheets/detail/dengue-and-severe-dengue. Accessed 23 Mar 2021

[129] Yauch LE, Shresta S (2008). Mouse models of dengue virus infection and disease. Antiviral Res.

[130] Yauch LE, Zellweger RM, Kotturi MF, Qutubuddin A, Sidney J, Peters B, Prestwood TR, Sette A, Shresta S (2009). A protective role for dengue virus-specific CD8+ T cells. J Immunol.

[131] Yoshida T, Omatsu T, Saito A, Katakai Y, Iwasaki Y, Kurosawa T, Hamano M, Higashino A, Nakamura S, Takasaki T, Yasutomi Y, Kurane I, Akari H (2013). Dynamics of cellular immune responses in the acute phase of dengue virus infection. Arch Virol.

[132] Yu CY, Chang TH, Liang JJ, Chiang RL, Lee YL, Liao CL, Lin YL (2012). Dengue virus targets the adaptor protein MITA to subvert host innate immunity. PLoS Pathog.

[133] Yuwono J, Suharyono W, Koiman I, Tsuchiya Y, Tagaya I (1984). Seroepidemiological survey on dengue and Japanese encephalitis virus infections in Asian monkeys. Southeast Asian J Trop Med Public Health.

[134] Zellweger RM, Prestwood TR, Shresta S (2010). Enhanced infection of liver sinusoidal endothelial cells in a mouse model of antibody-induced severe dengue disease. Cell Host Microbe.

[135] Zellweger RM, Eddy WE, Tang WW, Miller R, Shresta S (2014). CD8+ T cells prevent antigen-induced antibody-dependent enhancement of dengue disease in mice. J Immunol.

[136] Zellweger RM, Shresta S (2014). Mouse models to study dengue virus immunology and pathogenesis. Front Immunol.

[137] Zhang NN, Zhang L, Deng YQ, Feng Y, Ma F, Wang Q, Ye Q, Han Y, Sun X, Zhang FC, Qi X, Wang G, Dai J, Xia X, Qin CF (2019). Zika virus infection in Tupaia belangeri causes dermatological manifestations and confers protection against secondary infection. J Virol.

[138] Zheng Z, Li M, Liu Z, Jin X, Sun J (2020). Establishment of murine infection models with biological clones of dengue viruses derived from a single clinical viral isolate. Virol Sin.

[139] Zompi S, Harris E (2012). Animal models of dengue virus infection. Viruses.

[140] Zust R, Toh YX, Valdes I, Cerny D, Heinrich J, Hermida L, Marcos E, Guillen G, Kalinke U, Shi PY, Fink K (2014). Type I interferon signals in macrophages and dendritic cells control dengue virus infection: implications for a new mouse model to test dengue vaccines. J Virol.

